# Myocardial Infarction in a Patient With Homozygous Plasminogen Activator Inhibitor-1 (PAI-1) 4G/4G Mutation: A Case Report

**DOI:** 10.7759/cureus.95103

**Published:** 2025-10-21

**Authors:** AlMothana Manasrah, Farid Khan, Alon Yarkoni, Hisham Kashou, Afzal Rehman

**Affiliations:** 1 Department of Internal Medicine, United Health Services, Wilson Medical Center, Johnson City, USA; 2 Department of Cardiology, Heart and Vascular Institute, United Health Services, Wilson Medical Center, Johnson City, USA

**Keywords:** acute coronary syndrome, case report, hypercoagulability, myocardial infarction, plasminogen activator inhibitor-1, plasminogen activator inhibitor-1 4g/4g mutation

## Abstract

Non-ST-elevation acute coronary syndrome (NSTE-ACS) resulting from genetic thrombophilias such as plasminogen activator inhibitor-1 (PAI-1) polymorphisms is extremely uncommon. We report the case of a 47-year-old male with no significant past medical history, aside from marijuana use, who presented with chest pain and elevated troponin levels. Coronary angiography demonstrated a thrombotic 100% occlusion of the left circumflex artery. A hypercoagulability workup revealed a homozygous 4G/4G PAI-1 gene polymorphism, consistent with an increased thrombotic predisposition, with marijuana use serving as a possible trigger. The patient was treated with aspirin, clopidogrel, and apixaban. This case illustrates a rare association between myocardial infarction and the PAI-1 4G/4G polymorphism and underscores the importance of considering thrombophilia testing in young patients with ACS who lack conventional cardiovascular risk factors.

## Introduction

Acute coronary syndrome (ACS) is most commonly attributed to atherosclerotic plaque rupture [1]; however, non-atherosclerotic etiologies, including hypercoagulable states, coronary embolism, dissection, and vasospasm, have been reported to account for approximately 1%-13% of all ACS cases [2-5]. Based on studies by Shan et al. [6] and Badescu et al. [7], thrombophilic disorders such as antiphospholipid syndrome, Prothrombin G20210A, and factor V Leiden mutation have all been implicated in ACS.

Genetic thrombophilias, including mutations in factor V Leiden, prothrombin G20210A, methylenetetrahydrofolate reductase (MTHFR), and plasminogen activator inhibitor-1 (PAI-1), constitute important yet under-recognized causes of arterial and venous thrombosis [8-10]. The diagnostic workup for suspected hereditary thrombophilia generally includes evaluation for these variants in addition to testing for protein C, protein S, and antithrombin deficiencies, as well as antiphospholipid antibodies [11-13].

The PAI-1 gene encodes a key regulator of fibrinolysis that inhibits tissue- and urokinase-type plasminogen activators [13]. A common 4G/5G polymorphism in its promoter region affects gene expression, with the 4G allele associated with elevated PAI-1 levels and impaired fibrinolytic activity [14,15]. The homozygous 4G/4G genotype, present in roughly one-fifth of the population, has been linked to venous and, less reportedly, arterial thrombosis [16,17]. Only a few case reports have documented ACS related to this genetic variant, underscoring its potential as an independent prothrombotic mechanism [18-20]. We describe a case of non-ST-elevation acute coronary syndrome (NSTE-ACS) in a middle-aged man with homozygous PAI-1 4G/4G polymorphism, emphasizing the diagnostic evaluation, management, and clinical relevance of identifying genetic thrombophilia in patients without conventional cardiovascular risk factors.

## Case presentation

A 47-year-old Caucasian male with body mass index of 26 kg/m^2^ and no past medical history, aside from intermittent marijuana use, was evaluated for sudden onset pressure, like midsternal chest pain, radiating to the left arm and jaw, and worsening with exertion. He had no family history of premature coronary artery disease (CAD), sudden deaths, cardiovascular diseases, or hypercoagulable conditions such as venous thromboembolism or clotting disorders.

**Figure 1:**
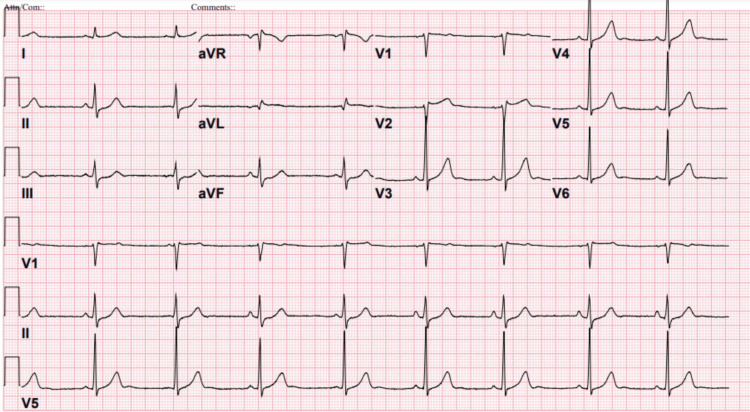
Electrocardiogram at admission demonstrating sinus bradycardia and incomplete right bundle branch block (RBBB).

The initial 12-lead ECG was notable for sinus bradycardia and incomplete right bundle branch block (RBBB) but did not demonstrate any ST-segment deviation, pathologic Q waves, or T-wave inversions (Figure 1). Repeat ECGs obtained after admission showed similar findings without dynamic ischemic changes. Initial laboratory workup was significant for an elevated troponin I level that peaked at 19.700 ng/mL (reference range: <0.012 ng/mL).

The patient was treated for NSTE-ACS with standard dual antiplatelets therapy (DAPT) including aspirin and ticagrelor and intravenous (IV) unfractionated heparin infusion. A transthoracic echocardiogram (TTE) confirmed normal biventricular function with a left ventricular ejection fraction of approximately 55% without wall motion impairment. Further laboratory evaluation, including a lipid panel, urine toxicology screen, thyroid-stimulating hormone, hemoglobin A1c, and D-dimer levels, was unremarkable (Table 1).

**Table 1:** Laboratory testing at the time of admission. TG: triglycerides, LDL-C: low-density lipoprotein cholesterol, HDL-C: high-density lipoprotein cholesterol, UTox: urine toxicology (screen), TSH: thyroid-stimulating hormone, HbA1c: hemoglobin A1c (glycated hemoglobin), D-DU: D-dimer units.

Test	Result	Reference Range
Lipid panel		
TG	32 mg/dL	<200 mg/dL
Total-C	129 mg/dL	<200 mg/dL
LDL-C	75 mg/dL	<100 mg/dL
HDL-C	48 mg/dL	>40 mg/dL
Non-HDL-C	81 mg/dL	60-100 mg/dL
UTox	Positive for cannabinoid otherwise negative including amphetamine and cocaine	
TSH	1.420	0.465-4.680 µIU/mL
HbA1c	5.2%	4.0%-5.6%
D-dimer	<150 ng/mL	≤243 ng/mL D-DU

**Figure 2:**
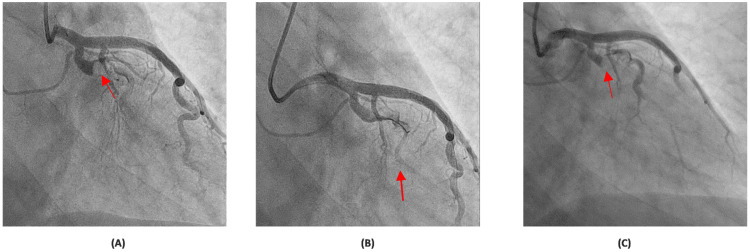
Coronary angiograms showing LCx occlusion and limited flow. CAG revealed a complete occlusion of the LCx and OM branch (red arrow) (A). Post wire manipulation and multiple balloon inflations showing only TIMI grade I flow achieved distally (red arrow) (B). Repeat CAG at 48 hours showing persistent proximal LCx occlusion with TIMI I flow in the OM branch (red arrow) (C). CAG: coronary angiography, LCx: left circumflex artery, OM: obtuse marginal, TIMI: Thrombolysis in Myocardial Infarction.

Subsequently, an invasive coronary angiography (CAG) was performed one day after presentation, revealing a 100% thrombotic occlusion of the large caliber left circumflex (LCx) and obtuse marginal (OM) with extensive clot burden in the entire LCx tree (Figure 2A). With wire manipulation, multiple balloon inflations, and intracoronary adenosine injections, only Thrombolysis In Myocardial Infarction (TIMI) I grade flow was established distally (Figure 2B). To reduce the thrombus burden, intravenous (IV) tirofiban, a glycoprotein IIb/IIIa inhibitor, was initiated in addition to dual antiplatelet therapy. A repeat CAG 48 hours later demonstrated persistent 100% occlusion of the proximal LCx with TIMI I grade flow in the OM branch (Figure 2C). Percutaneous coronary intervention (PCI) was not pursued due to significant and persistent clot burden. Surgical revascularization was not considered given the isolated nature of the lesion and the absence of multivessel disease.

The patient was discharged on triple antithrombotic therapy consisting of aspirin, clopidogrel, and apixaban. The decision to include apixaban, in addition to dual antiplatelet therapy, was based on the extensive intracoronary thrombus burden and the presumed hypercoagulable etiology. He was also prescribed a high-intensity statin. Beta-blocker and angiotensin-converting enzyme inhibitors/angiotensin II receptor blockers were deferred at discharge due to persistent sinus bradycardia and borderline low blood pressure.

Extensive thrombophilia workup was performed that included lipoprotein-(a), factor V Leiden, cardiolipin antibodies, methylenetetrahydrofolate reductase (MTHFR) gene mutation, homocysteine, fibrinogen, protein C and S, antithrombin activity, lupus anticoagulant, phospholipid autoantibodies, prothrombin G20210A mutation, and PAI-1 gene polymorphism as given in Table 2. 

**Table 2:** Thrombophilia workup. MTHFR: methylenetetrahydrofolate reductase gene mutation, PAI-1: plasminogen activator inhibitor-1, MPL: M-phospholipid units, GPL: G-phospholipid units.

Test	Result	Reference Range/Interpretation
Lipoprotein-(a)	15 nmol/L	<75 nmol/L
Antithrombin	88%	80%-130%
Factor V Leiden	Negative	Negative
MTHFR Mutation	Heterozygous for the C677T variant and negative (normal) for the A1298C variant in the MTHFR gene	This result is not associated with a significantly increased risk for the coronary artery disease, venous thromboembolism, or adverse pregnancy outcome
Homocysteine	10.88 µmol/L	5.00-15.00 µmol/L
Fibrinogen	379.0 mg/dL	200.0-393.0 mg/dL
Protein C	96%	72%-160%
Protein S Activity	94%	65%-150%
Lupus anticoagulant	Negative	Negative
Phospholipid autoantibodies		
IgM	<9.4 MPL	<15.0 (Negative) MPL
IgG	<9.4 GPL	<15.0 (Negative) GPL
Prothrombin G20210A mutation	Negative	Negative
PAI-1 gene polymorphism	4G/4G	Homozygous for the 4G deletion allele

Hypercoagulability studies were negative for the most common disorders; however, the analysis of PAI-1 promoter polymorphism revealed the presence of homozygous 4G/4G genotype. On outpatient follow-up, he denied any recurrence of chest pain or new cardiac symptoms. He was followed in the outpatient setting by cardiology, hematology, and genetic counseling services, with recommendations for family screening. He was also extensively counseled on abstaining from marijuana use to minimize future thromboembolic risk.

## Discussion

ACS resulting from coronary thromboembolism was first reported in the 19th century [3]. Coronary thromboembolism is suspected as the likely culprit for ACS when CAG shows the presence of a thrombus, appearing as a noncalcified filling defect surrounded by contrast on at least three sides, in the absence of significant atherosclerotic disease [4]. The latter is defined as the lack of luminal narrowing exceeding 25% throughout the coronary tree [4]. In our patient, the absence of atherosclerotic disease in addition to the high thrombus burden suggests that the thromboembolic process was the most likely etiology of ACS.

The thromboembolic process leading to coronary embolism has been well described in the literature. Direct emboli from cardiac structures such as left atrial appendage or valves, intracardiac thrombi or neoplastic material, paradoxical emboli from venous thrombi via intracardiac or pulmonary shunts, and iatrogenic emboli primarily occur after invasive valvular or coronary interventions [21,22]. In our case, the absence of these etiologies along with the absence of atherosclerotic CAD in a healthy young patient raised suspicion for hypercoagulability disorder. He tested negative for the most common thrombophilia disorders apart from a rare genetic mutation of PAI-1 promoter polymorphism linked to thromboembolism.

PAI-1, also known as SERPINE-1, is a key serine protease inhibitor that regulates the fibrinolytic system, which breaks down blood clots and prevents thrombosis [23,24]. PAI-1 controls the activity of tissue-type plasminogen activator (tPA) and urokinase-type plasminogen activator (uPA), enzymes that convert plasminogen into plasmin, the enzyme responsible for fibrin degradation [23,24]. Studies of the 5G/4G polymorphism have demonstrated higher plasma PAI activity in individuals with the 4G allele compared to those with the 5G allele in ACS patients [15,16,25,26]. This may be attributed to the 5G allele having an additional binding site for a repressor, which results in lower transcription rates and reduced PAI-1 activity [15,16,26]. In our patient, PAI-1 promoter polymorphism was evaluated by using polymerase chain reaction (PCR) and restriction fragment length polymorphism (RFLP). The 4G/4G genotype was identified, which is associated with PAI-1 levels approximately 25% higher compared to individuals with either the 5G/5G or 4G/5G genotype [27], making our patient more susceptible to coronary thrombosis. Parpugga et al. demonstrated that carriers of the 4G allele, particularly those with the 4G/4G genotype, had significantly higher PAI-1 levels and a greater likelihood of complete coronary artery occlusion during myocardial infarction compared with 5G/5G carriers [28]. This study supports the mechanistic basis of our case, where impaired fibrinolysis due to the 4G/4G genotype likely contributed to the extensive intracoronary thrombus and complete occlusion observed despite the absence of atherosclerosis [28].

Virchow’s triad describes three fundamental pathological mechanisms contributing to thrombus formation: hypercoagulability, alterations in blood flow, and endothelial injury or vessel wall damage [29]. In this patient, marijuana use was likely the inciting factor contributing to endothelial injury, as multiple reports have linked marijuana consumption to impaired endothelial function [30] and an increased risk of ACS [31]. This effect is thought to be mediated through oxidative stress, inflammation, and increased production of reactive oxygen species, leading to reduced nitric oxide bioavailability and endothelial-dependent vasodilation. Additionally, cannabinoids may promote platelet activation and microvascular dysfunction, further contributing to a prothrombotic state [32].

We reason that our patient had elevated natural predisposition to coronary thrombosis due to homozygous PAI-1 4G/4G polymorphism. Accompanying use of marijuana might have conferred an even increased risk for thrombotic phenomenon causing endothelial damage and triggering thrombotic process and resultant occlusive MI. A few cases have described ACS events associated with PAI-1 gene polymorphisms [18-20]. Among hereditary thrombophilias, factor V Leiden (prevalence ~3%-8%) and prothrombin G20210A mutation (~1%-3%) are the most common, whereas PAI-1 4G/4G polymorphism and MTHFR mutations are considered less frequent and are typically tested when common thrombophilic disorders are excluded [15,33-35]. Testing rare hypercoagulability genetic disorders may be helpful in the absence of common thrombophilia disorders and atherosclerotic disease and risk factors.

A systematic review analyzing 147 documented cases of coronary thromboembolism identified various treatment approaches, including thrombectomy, thrombolysis, balloon angioplasty, and stent placement [36]. In our patient, PCI was attempted with multiple balloon inflations but was unsuccessful. Mechanical thrombectomy or thrombus aspiration was not performed due to the extensive thrombus burden and the associated risk of distal embolization and no-reflow phenomenon. The decision against further intervention was based on the overall low likelihood of successful reperfusion and the potential procedural complications, particularly stent thrombosis [37].

TIMI flow grade is a standardized angiographic classification used to assess coronary blood flow after reperfusion therapy. TIMI grade I indicates penetration without perfusion, meaning there is faint opacification of the distal coronary bed but without full distal vessel filling. In contrast, TIMI grade III represents normal flow, with complete filling and clearance of contrast comparable to an uninvolved artery [38]. In our patient, the post-intervention CAG demonstrated TIMI grade I flow. This finding reflects a severe residual thrombotic burden.

There are no established guidelines for the management of PAI-1-induced MI. A triple antithrombotic therapy was based on the rationale that thromboembolic conditions are generally managed with anticoagulation [39]. Additionally, platelets are a major source of circulating PAI-1 and clinical data suggest that long-term antiplatelet therapy, such as aspirin and P2Y12 inhibitor, can effectively reduce plasma PAI-1 levels [40].

## Conclusions

PAI-1 promoter gene mutation may lead to increased thromboembolic risk with potential coronary thrombosis or embolism that may present like an occlusive MI. Keeping a low threshold for testing rare hypercoagulability genetic disorders may be helpful in the absence of common thrombophilia disorders and atherosclerotic disease or its risk factors.
